# Determination of the Young's modulus of the epicuticle of the smooth adhesive organs of *Carausius morosus* using tensile testing

**DOI:** 10.1242/jeb.105114

**Published:** 2014-10-15

**Authors:** Michael Bennemann, Stefan Backhaus, Ingo Scholz, Daesung Park, Joachim Mayer, Werner Baumgartner

**Affiliations:** 1 Department Cellular Neurobionics, Institute for Biology II, RWTH Aachen University, Worringerweg 3, 52074 Aachen, Germany; 2 Westfälisches Institut für Bionik, Westfälische Hochschule Gelsenkirchen Bocholt Recklinghausen, Münsterstraße 265, 46397 Bocholt, Germany; 3 Central Facility for Electron Microscopy, RWTH Aachen University, Ahornstraße 55, 52074 Aachen, Germany; 4 Institute of Biomedical Mechatronics, University of Linz, Altenbergerstraße 69, 4040 Linz, Austria

**Keywords:** Finite element simulation, Cuticle, Adhesion, Stick insects, Arolium, *Carausius morosus*

## Abstract

Adhesive organs like arolia of insects allow these animals to climb on different substrates by creating high adhesion forces. According to the Dahlquist criterion, adhesive organs must be very soft, exhibiting an effective Young's modulus of below 100 kPa to adhere well to substrates. Such a low effective Young's modulus allows the adhesive organs to make almost direct contact with the substrate and results in van der Waals forces along with capillary forces. In previous studies, the effective Young's moduli of adhesive organs were determined using indentation tests, revealing their structure to be very soft. However, adhesive organs show a layered structure, thus the measured values comprise the effective Young's moduli of several layers of the adhesive organs. In this study, a new approach is illustrated to measure the Young's modulus of the outermost layer of the arolium, i.e. of the epicuticle, of the stick insect *Carausius morosus*. As a result of the epicuticle being supported by upright fibres, tensile tests allow the determination of the Young's modulus of the epicuticle with hardly influence from subjacent layers. In our tensile tests, arolia of stick insects adhering on a latex membrane were stretched by stretching the membrane while the elongation of the contact area between an arolium and the membrane was recorded. For analysis, mathematical models of the mechanical system were developed. When fed with the observed elongations, these models yield estimates for the Young's modulus of the epicuticle of approximately 100 MPa. Thus, in arolia, a very thin layer (~225 nm) of a rather stiff material, which is less susceptible to abrasion, makes contact with the substrates, whereas the inner fibrous structure of arolia is responsible for their softness.

## INTRODUCTION

Many insects possess specialised attachment organs on their tarsi, which enable them to climb and even walk upside down on almost all substrates. Some species are even capable of resisting extreme pull-off and shear forces equivalent to more than 100 times their own body weight (Eisner and Aneshansley, 2000; Federle et al., 2000), and to move with a high velocity. The detailed underlying mechanisms of this impressive performance are still unclear, but they are presumably based on a combination of a complex ultrastructure and the physical properties of the adhesive organs.

Adhesive organs in arthropods and vertebrates have been classified into ‘smooth’ pads with a soft cuticle (e.g. arolia) and ‘hairy’ pads (Beutel and Gorb, 2001). The cuticle of the arolium of *Carausius morosus* Sinéty 1901 differs structurally from typical hard exoskeleton cuticles and from the soft and flexible cuticles found in joints and extensible body parts. In arolia, the fibres in the procuticle are neither orientated parallel nor perpendicular to the surface, but they are oriented in a specific angle to it (Scholz et al., 2008; Bennemann et al., 2011).

Adhesion between smooth pads and a surface is mainly determined by two forces: capillarity forces caused by a liquid layer (‘wet adhesion’) and van der Waals forces. Van der Waals forces are only achieved at nearly direct contact between pad and surface (the liquid layer has to be <10 nm) (Kendall, 2001; Dirks and Federle, 2011). For such close contact, adhesive organs have to be very resilient.

The adhesion performance of arthropods and vertebrates has aroused high interest in biologists and engineers trying to create artificial reusable adhesion devices (Gorb et al., 2007; Shen et al., 2008; Röhrig et al., 2012). To transfer the functionality of adhesive organs into technical devices, it is important to determine the mechanical properties of the materials from which adhesive organs are composed. Until now, the mechanical properties of only a few materials in adhesive organs were known (Peattie et al., 2007; Bullock and Federle, 2009). In previous studies, analyses of the mechanical properties of smooth adhesive organs have been performed using indentation tests (Gorb et al., 2000; Perez Goodwyn et al., 2006; Scholz et al., 2008; Scholz et al., 2009; Barnes et al., 2011). Indentation tests of layered structures have the disadvantage that subjacent layers – and in the case of adhesive organs also their hydroflation, i.e. their filling with liquid – have an impact on the measurement of superficial layers. This means that in indentation tests not only the Young's modulus of the layer, which was indented, is determined, but the effective Young's modulus of the uppermost and several layers below is also measured. Simplified, in indentation tests, the effective Young's modulus of all layers down to approximately tenfold of the indentation depth is measured. In previous studies on the Young's moduli of the adhesive organs of *C. morosus* and *Litoria caerulea* (Scholz et al., 2008, Scholz et al., 2009), an indentation depth of approximately 1 μm led to an effective Young's modulus for the combination of cuticulin,

**List of symbols and abbreviations**

*A*: cross sectional area
CGI: cuticle-geometry one
CGII: cuticle-geometry two
CPD: critical point dryer
*E*: Young's modulus
*F*: force
FIB: focused ion beam
*l*_0_: length of the membrane at the beginning of the tensile test
PBS: phosphate buffered saline
Δ*L*: elongation of the membrane

epicuticle and procuticle layer in *C. morosus* and to an effective Young's modulus of the uppermost cell layers in *L. caerulae*.

In this study, we are especially interested in the Young's modulus of the outermost layer of the arolium, the so called epicuticle, which contacts the substrate and mediates the adhesion. To determine the Young's modulus of the epicuticle, we used tensile tests, in which forces are acting almost exclusively on the outermost layer of the arolia. A further advantage of tensile tests is that the Young's modulus can be determined in different orientations. This is important for the measurement of anisotropic structures, such as the adhesive organs of stick insects (Scholz et al., 2008).

The aim of our study was to characterise the detailed ultrastructure responsible for the mechanical properties of the arolium of *C. morosus* and to develop a new method to determine the Young's modulus of soft superficial biological membranes, such as the epicuticle, with less impact from the underlying layers as present in indentation tests.

## RESULTS

### Ultrastructure of the arolium

**Fig. 1.**
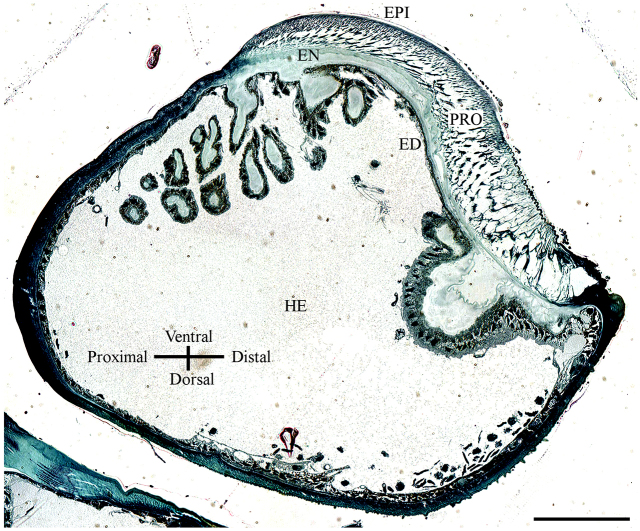
**Light microscopic image of a semi-thin longitudinal section through the pretarsus.** The layered construction of the arolium cuticle is clearly visible. EPI, epicuticle; PRO, procuticle; EN, endocuticle; ED, epidermis; HE, hemolymph. Scale bar: 100 μm.

**Fig. 2.**
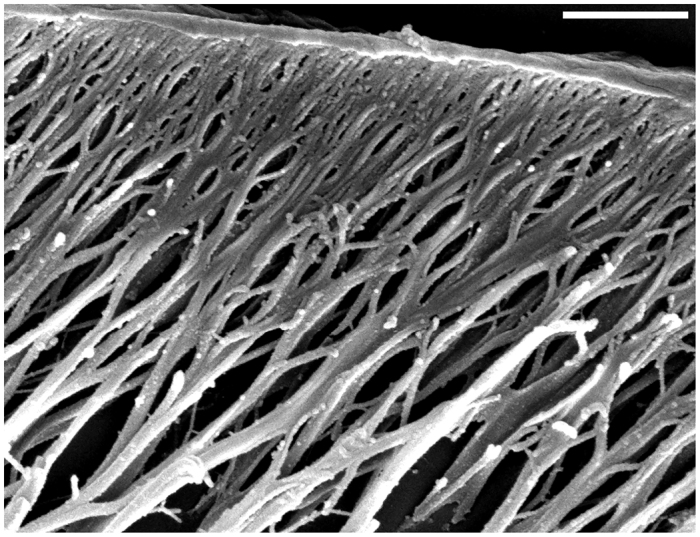
**Final branching of the fibres in the procuticle.** Scanning electron microscopic image of a semi-thin sagittal section from which the epoxy resin was etched off. The thinnest branches of the fibres in the procuticle and their connection to the adjacent epicuticle can be seen. Scale bar: 2 μm.

**Fig. 3.**
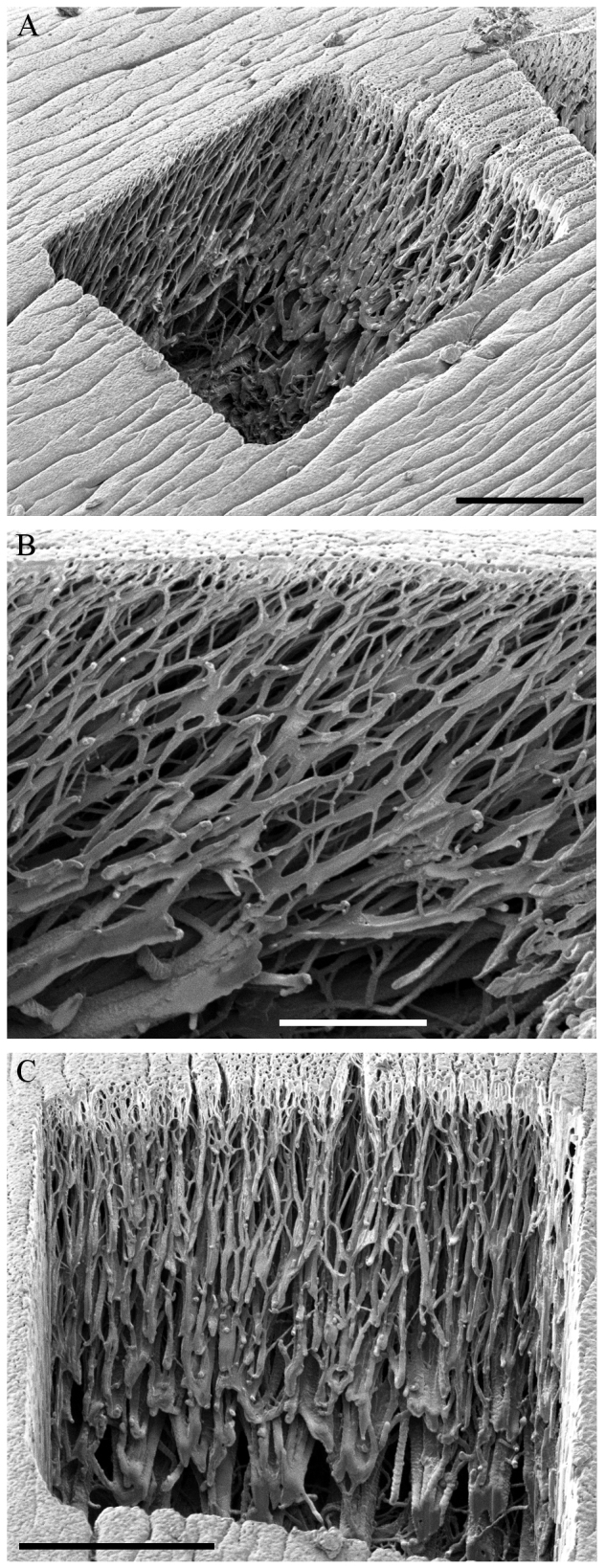
**Secondary electron images of the fibrous inner structure of the arolium.** (A) Overview of a rectangular cut into an arolium prepared by focused ion beam (FIB). (B) Fibrous structure in the proximal to distal orientation. The fibres are orientated in an angle of approximately 57 deg to the epicuticle. (C) Fibrous structure in the left to right orientation. The fibres are orientated perpendicular to the epicuticle. Bundles of fibres ramify into progressively finer fibres. The image was taken in an angle of 82 deg to the cutting edge. Scale bars: 10 μm (A), 5 μm (B), 10 μm (C).

To understand the functional mechanics of the arolium, its inner three-dimensional (3D) structure had to be determined. Fig. 1 shows an image of a longitudinal semi-thin section through the entire pretarsus, obtained using light microscopy. Clearly, a layered structure of the cuticle can be seen. The outermost epicuticle, the fibrous procuticle, the subjacent endocuticle, the underlying epidermis and a hemolymph filled region can be distinguished (Fig. 1). The epicuticle is a thin membrane of ~225 nm thickness (Fig. 2). The fibres in the procuticle originate at the subjacent endocuticle and branch in a tree-like manner into fine fibres connected to the epicuticle (Fig. 2). To determine the exact orientation of the fibres, a 3D focused ion beam (FIB) cutting of the arolium was performed. The now accessible structures can be seen in Fig. 3. In the proximal to distal orientation, the fibres are arranged at an angle of approximately 57 deg to the epicuticle (Fig. 3B) and, in the left to right orientation, the fibres are orientated perpendicularly to the epicuticle (Fig. 3C). In the left to right orientation, it is visible that bundles of fibres ramify into progressively finer fibres (Fig. 3C). In both orientations, the fibres are connected by only a few crosslinks, which suggests that the individual branches cannot be moved completely independently. The thin epicuticle exhibits some longitudinal grooves. Below these grooves, none of the finest fibres are located (Fig. 3C). These findings are in line with results obtained using alternative approaches, such as freeze fractioning or etching methods (data not shown), which yielded, in principle, the same results. Finding the same ultrastructure using different techniques confirmed that the observed structures represent the *in vivo* situation and not confounding factors from the preparation methods. We focus here on the described method, because the other methods suffer from the disadvantages that it is difficult to analyse the ultrastructure from a particular perspective and that simultaneous observation of the longitudinal and transversal planes is not possible for a single specimen.

### Young's modulus of the epicuticle

**Fig. 4.**
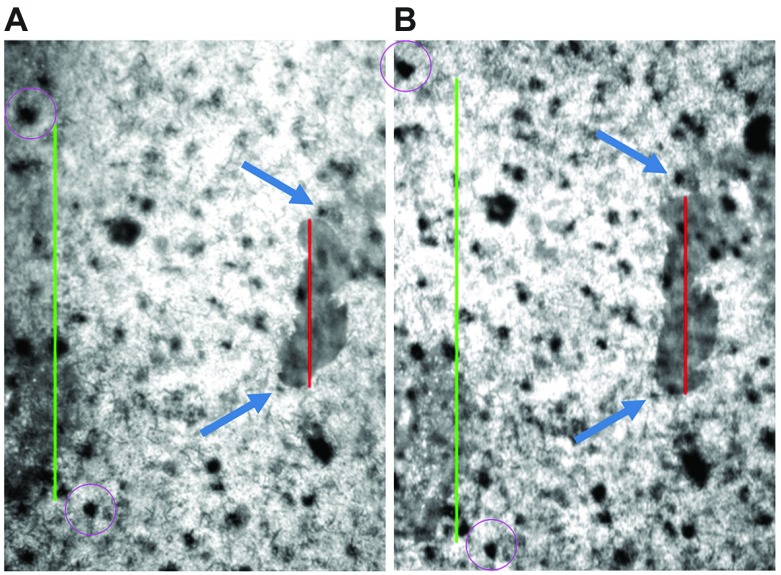
**Epi-illumination images of the tensile test ‘transversal 3’.** (A) Image at the beginning and (B) end of the stretching. In both images, the contact area of the arolium to the latex membrane is visible (dark grey). The red lines indicate the distances measured to determine the elongation of the width of the arolium contact area, and the green lines indicate the distances measured to determine the elongation of the latex membrane. The green lines are defined by two reference points on the latex membrane (encircled in violet). The contact area did not slide on the latex membrane, visible by the position relative to the two reference points highlighted by blue arrows. The red line in image A measures 208 μm.

To determine the Young's modulus of the thin epicuticle, tensile tests were performed. To achieve this, stick insects were allowed to adhere onto a thin latex membrane, which was stretched while simultaneously observing the elongation of latex membrane and arolium contact area under a microscope. Tensile tests in which the cuticle was stretched parallel to the width of the arolium (i.e. left to right) are denoted from here on as transversal tensile tests, and tensile tests in which the cuticle was stretched parallel to the length

of the arolium (i.e. distal to proximal) are denoted from here on as longitudinal tensile tests. To determine the elongation of the contact area and the latex membrane during transversal or longitudinal tensile tests, the length of the contact area and the distance between two reference points on the latex membrane were measured at the beginning and end of the tensile tests (Fig. 4) and at two time points in between. Eleven transversal tensile tests were included in the evaluation (Table 1). In all of these tensile tests, the contact area of the arolium did not change its position relative to the reference points on the latex membrane. Furthermore, no structures of the latex membrane, which had been under the arolium contact area at the beginning of the stretching, appeared next to the arolium contact area during stretching (Fig. 4). This means that the arolium did not slide during the experiments. Furthermore, there were no large changes in the angle between the tarsi and the contact areas during stretching, and no large changes of the contours of the arolium contact areas occurred during stretching. If slipping, turning or active movement of a stick insect occurred, the corresponding experiments were excluded from the evaluation.

In the transversal direction, the procuticle fibres were not stretched but bent, thus hardly influencing the force needed to stretch the arolium, whereas the stretching of the epicuticle yielded the highest impact.

In the transversal tensile tests, the latex membrane was stretched by up to 121.8% [±3.3% (s.d.), *N*=11, Table 1] of the initial length, resulting in a coupled, perfectly linear stretching of the arolium when the latex membrane was stretched more than approximately 10% of the total stretching of 21.8%. At elongations less than 10%, the epicuticle exhibited a smaller apparent stiffness, most probably representing the flattening of the grooves seen in the unstretched arolium. At higher levels of strain, the behaviour was linear, indicating a homogeneous linear elastic material. At full expansion of the latex membrane (121.8%), the cuticle adhering on the latex membrane was stretched to approximately 112.0% (±3.7%, *N*=11, Table 1), which corresponded to an elongation of 55.8% (±14.9%, *N*=11, Table 1) of the elongation of the latex membrane.

In longitudinal tensile tests, the arolium contact area increased only very little or not at all in length, and often the orientation of the tarsus to the contact area changed greatly, resulting in a deformed

contact area. These findings are in line with the ultrastructure, because, owing to the angular orientation of the fibres in the procuticle, a stretching of these fibres would be necessary during a stretching of the arolium in the longitudinal direction. Therefore, we refrained from determining the Young's modulus of the epicuticle in the longitudinal direction.

The latex membrane was initially characterised and found to exhibit a thickness of 60 μm and a Young's modulus of 1.2 MPa (±0.008 MPa, *N*=3, in supplementary material Table S1; Fig. S1).

These values in combination with the observed linearity of the elongations of the arolium and the latex membrane allow for a rough estimation of the Young's modulus of the arolium: assuming both materials have a linear elasticity, and neglecting the influence of the layers subjacent to the epicuticle and imperfections of the strain transmission due to geometric restrictions, one can calculate

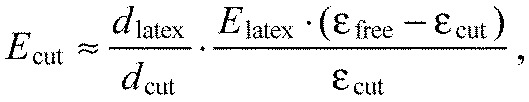

with *E*_cut_ being the Young's modulus of the epicuticle, *d*_latex_ the thickness of the latex membrane (=60 μm), *d*_cut_ the thickness of the epicuticle (=0.4 μm), *E*_latex_ being the Young's modulus of the latex membrane (=1.2 MPa), ε_free_ being the strain (relative elongation) of the free latex membrane (=0.208, transversal test 4) and ε_cut_ being the strain in the contact zone between the latex membrane and arolium (=0.106, transversal test 4). Using the example of the tensile test transversal four, this yields a Young's modulus of *E*_cut_=173.2 MPa.

**Fig. 5.**
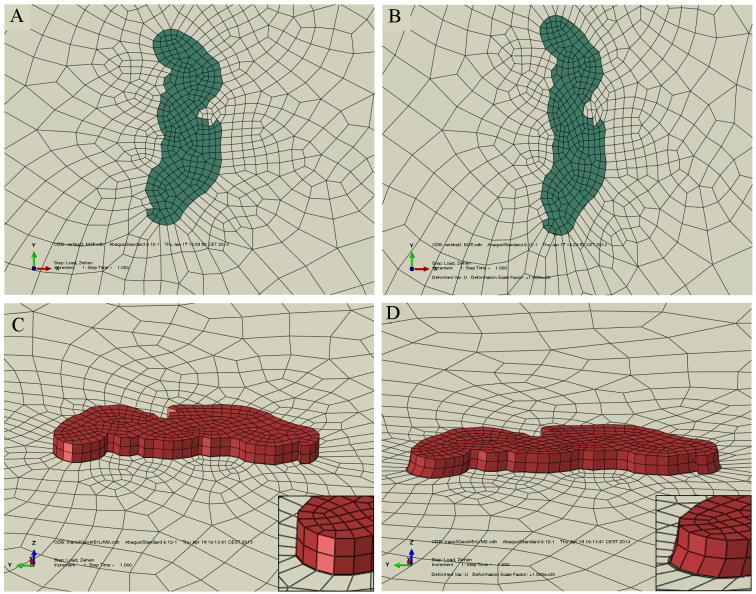
**Images of the finite element simulations of the tensile test transversal 3 using CGI and CGII.** (A,B) CGI (top view), (C, D) CGII (side view), (A,C) non-deformed cuticle (green and red) on a latex membrane (grey), (B,D) deformed cuticle after stretching the latex membrane approximately 23.2%, and the adjustment of the Young's modulus of the cuticle to B, 34.5 MPa; D, 2 MPa. The insets in C and D show details of the cuticle geometry. In C and D, the surroundings of CGII are hidden to show the deformation of the thin epicuticle layer.

To take into account the effects of the layers subjacent to the epicuticle and of the complicated geometry of the arolium contact area, finite element simulations were performed adjusting the Young's modulus of the epicuticle to fit the experimental observations. Two different models were used. The first, cuticle-geometry one (CGI), comprised only the thin epicuticle showing a uniform Young's modulus and the contour of the arolium contact area. The second, cuticle-geometry two (CGII), consisted of the epicuticle supported by a thicker, soft layer comprising the layers subjacent to the epicuticle and both being surrounded by the cuticle of the non-contacting areas of the arolium. These two models comprise the extreme cases, and the exact Young's modulus should be somewhere in between the found values. The finite element simulations for the first four transversal tensile tests resulted in the following Young's moduli: the Young's modulus of the epicuticles with CGI had to be adjusted on 173.4 MPa (±135.8 MPa, *N*=4, Fig. 5; Table 2) and the epicuticles with CGII had to be adjusted on 27.3 MPa (±26.2 MPa, *N*=4, Fig. 5; Table 2).

The finite element simulations of the first four tensile tests showed for both models a linear relationship between the relative percentage elongations of the epicuticle (percentage elongation of the epicuticle divided by the percentage elongation of the latex membrane) in relation to the determined Young's moduli (supplementary material Fig. S2). This is in line with the experimental observations and allowed the determination of the Young's moduli of the seven further tensile tests based on the results of the finite elements simulations of the first four tensile tests. This estimation resulted in the Young's moduli denoted in Table 2.

**Fig. 6.**
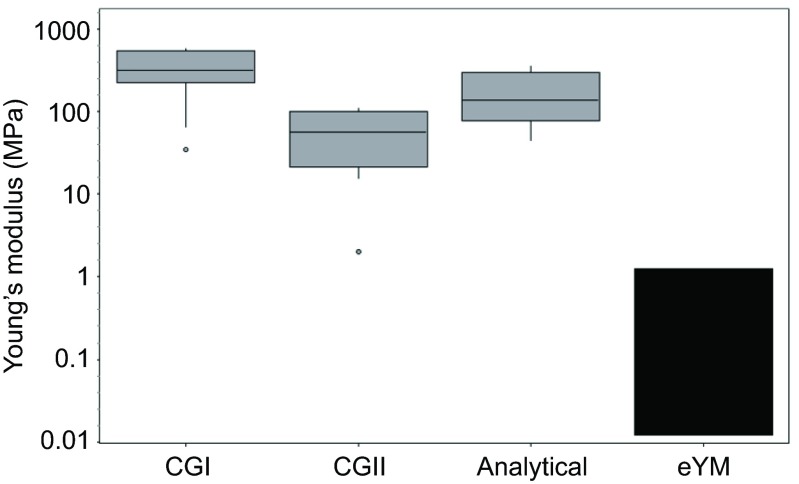
**Young's moduli of the epicuticle in comparison to the effective Young's modulus of adhesive organs.** The first three boxes show the Young's moduli of the epicuticle, as determined by tensile testing followed by finite element simulation (CGI, CGII) or analytical determination (Analytical). The ends of the boxes define the 25th and 75th percentiles, with a line at the median and error bars to the maximum and minimum values (without regard to outliers). Outliers were marked as points and are defined as Z_u_=Q_1_−1.5*(Q_3_−Q_1_); Z_o_=Q_3_+1.5*(Q_3_−Q_1_) with Z_u_=outlier bottom, Z_o_=outlier top, Q_1_=25% quartile, Q_3_=75% quartile. The box furthest to the right shows the effective Young's moduli (eYM) of whole adhesive organs, as determined by indentation testing. These Young's moduli were adopted from Gorb et al. (Gorb et al., 2000), Perez Goodwyn et al. (Perez Goodwyn et al., 2006) and Scholz et al. (Scholz et al., 2008). The Young's modulus is plotted logarithmically.

Combining the Young's moduli determined by finite element simulation and the Young's moduli estimated by using the results of the finite element simulations resulted in the following Young's moduli for the different cuticle geometries (Fig. 6; Table 2): CGI, 329.9 MPa (±174.9 MPa, *N*=11); CGII, 58.7 MPa (±34.8 MPa, *N*=11). The simple analytical approximation derived above yielded 168.6 MPa (±106.4 MPa, *N*=11).

## DISCUSSION

Adhesive organs of climbing animals exhibit remarkable abilities with respect to adhesion and friction force generation in combination with rapid detachment. In order to adhere well onto different substrates, adhesive organs need to fulfil different requirements. According to the Dahlquist criterion (Dahlquist, 1966), materials are tacky when their Young's modulus is lower than approximately 100 kPa. The overall Young's moduli of adhesive organs from different species, i.e. the stress–strain relationship of their whole composite structure, were found previously to exhibit such low stiffnesses through characterising their Young's moduli using indentation tests (Gorb et al., 2000; Perez Goodwyn et al., 2006; Scholz et al., 2008; Scholz et al., 2009; Barnes et al., 2011). However, if adhesive organs consisted of a material with a Young's modulus of 100 kPa or below, they would be very susceptible to wear. We assume that such a low effective modulus of the whole structure can still be achieved when the adhesive organs are covered with a thin membrane with a high Young's modulus, so long as the subjacent structure shows a high softness. Such adhesive organs consisting of a soft core covered with a stiffer membrane combine conformability to the surface roughness with resilience to the

**Table 1.**
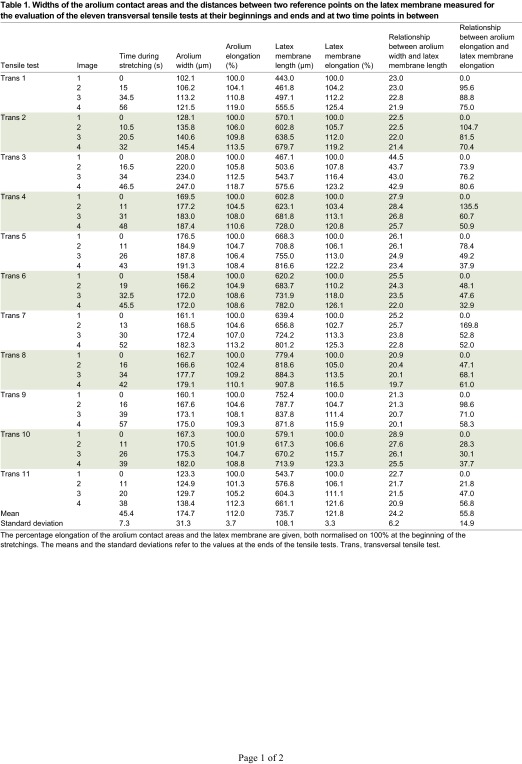
Widths of the arolium contact areas and the distances between two reference points on the latex membrane measured for the evaluation of the eleven transversal tensile tests at their beginnings and ends and at two time points in between

environment (Perez Goodwyn et al., 2006). This still allows adhesive organs to conform to surface asperities.

In the case of animal adhesion between smooth adhesive organs and a surface, a further factor has to be taken into account. An ‘adhesive liquid’ produced by animals causes capillary forces and increases adhesion forces in most cases (Dixon et al., 1990; Drechsler and Federle, 2006; Edwards and Tarkanian, 1970). Capillary forces play a major role in adhesion to rough surfaces, when surface cavities are filled up with the liquid. If the layer of liquid gets too thick, friction is reduced rapidly and adhesion is lost. To minimise the liquid layer between adhesive organ and substrate, the adhesive organ has to adapt very closely to the substrate, requiring a biological material with a low Young's modulus (Drechsler and Federle, 2006; Federle et al., 2006; You and Wan, 2013). Despite the wet adhesion realised in the adhesive organs of stick insects in the evaluated tensile tests, we did not find evidence for the arolium slipping on the latex membrane. This might be founded in the special non-Newtonian properties of the adhesive liquid and the very thin fluid layer, which let the adhesive liquid act as a solid at lower forces (Drechsler and Federle, 2006; Dirks et al., 2010).

A thin coverage can better conform to rough substrates than a thick coverage of the same material, when the thickness of the thin

coverage is smaller than the wavelength of the roughness of the substrate (Persson and Gorb, 2003; Gorb, 2008). Transferred to *C. morosus*, whose epicuticle has a thickness of approximately 225 nm, it should be able to adhere to substrates with asperities, which are from bottom to top higher than 225 nm, only by passively folding its epicuticle around surface asperities during attachment. This conformation to the substrate might be supported by the fluid inside the cuticle, which enables the membrane to follow the contours of the substrate to which the insect is adhering. For folding or bending, a much higher Young's modulus is tolerable than for elastic Hertz-deformation of a semi-infinite material. For biomimicry this means that it is favourable to produce artificial adhesion devices with preferable thin terminating membranes of a higher Young's modulus, which are supported by an underlying layer of lower stiffness.

The usage of tensile testing in place of indentation testing enabled the determination of the Young's modulus of the epicuticle itself, instead of the effective Young's modulus of several layers of the arolium. Scholz et al. (Scholz et al., 2008) assumed that the stiffness of the adhesive organs of stick insects increased with increasing depth of the cuticle. If this were the case, the fibrous procuticle would show a higher stiffness than the epicuticle. Especially in the direction parallel to the surface of the adhesive organs, this is unlikely because the fibres in the procuticle are orientated almost perpendicular to this direction and therefore cannot absorb tensile forces.

In indentation tests, the hydroflation of the pretarsus and, depending on the indentation depth, several layers of the cuticle have an impact on the deformation behaviour of the adhesive organ, meaning that only the effective Young's modulus of several layers can be determined. In previous studies of stick insects (Scholz et al., 2008), an indentation of approximately 1 μm using a sharp atomic force microscopy (AFM) cantilever is influenced not only by the epicuticle and outermost wax layer, but also by the subjacent procuticle layer. In tensile testing, the hydroflation of the pretarsus is negligible because mainly the outer layers of the arolium have an impact on the deformation behaviour.

During the transversal tensile tests, first, the cuticle might be not stretched but only spread until the epicuticle no longer showed grooves, which are normally present on the surface of the arolia. This is consistent with the observation that the cuticle was elongated more at the beginning of the tensile tests than at the end (Table 1). Based on the great extent of elongation of the epicuticle of 12%, it is safe to assume that the initial spreading of the epicuticle is negligible for the determination of the Young's modulus. The fact that the cuticle was stretched more at the beginning of the tensile tests indicates that the fibres in the procuticle do not have a high impact on the force needed to stretch the epicuticle in the transversal direction. The force needed to bend the fibres should be consistent from the beginning of the stretching. Assuming that the fibres have

**Table 2.**
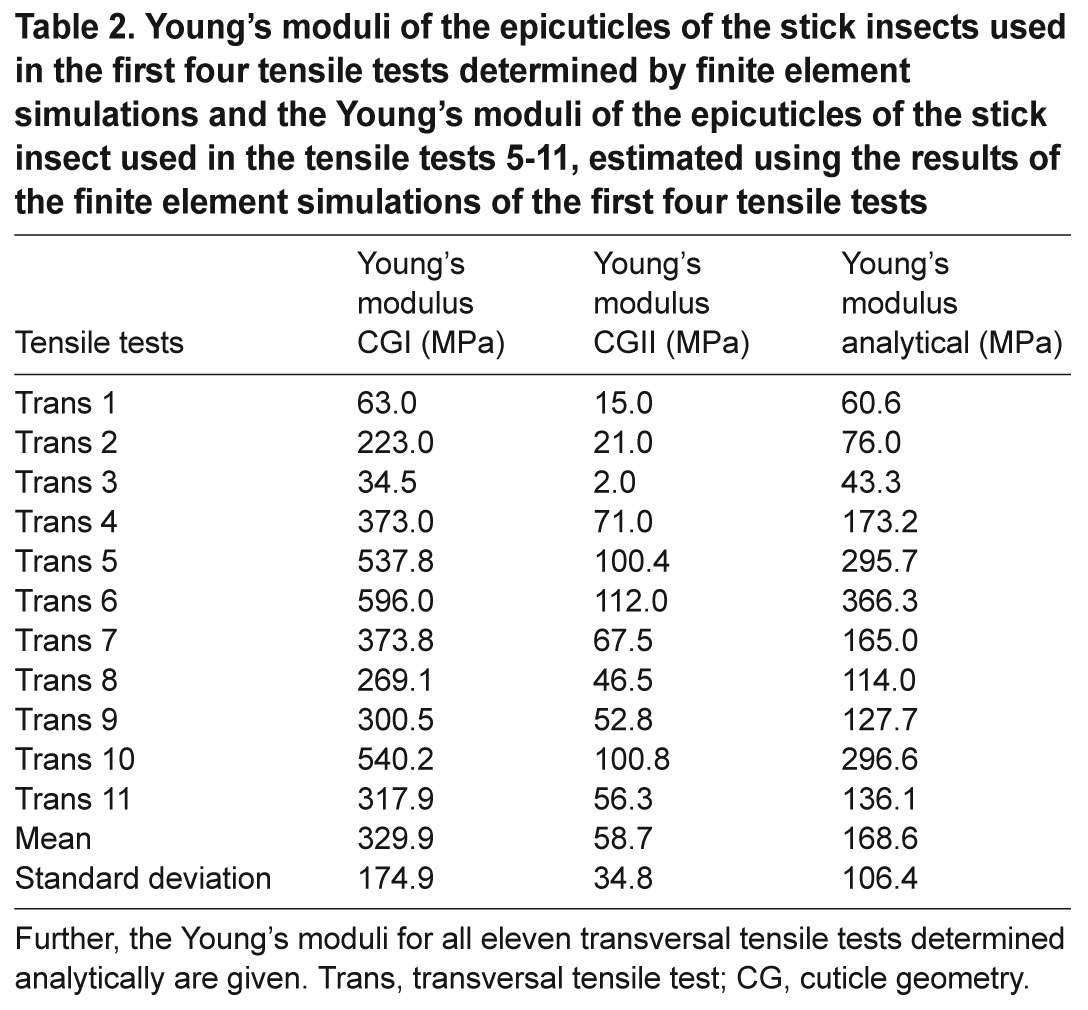
Young's moduli of the epicuticles of the stick insects used in the first four tensile tests determined by finite element simulations and the Young's moduli of the epicuticles of the stick insect used in the tensile tests 5-11, estimated using the results of the finite element simulations of the first four tensile tests

a low impact on the tensile rigidity of the cuticle supports the high Young's moduli that were determined for the epicuticle through tensile testing.

For the reasons listed above, the Young's moduli determined in this study are much higher than the effective Young's moduli that have been determined in previous studies (Gorb et al., 2000; Scholz et al., 2008). It has to be emphasised that this is a very robust result. Although we and others have found effective Young's moduli of several kilopascal by indentation testing in the past, the sound value for the epicuticle is in the range of several to a few hundred megapascal. Even if there are some parameters that have not been investigated here and might influence the findings, such as the exact

thickness of the cuticle layers, the influence of the surrounding tissue or the Poisson's ratio of the materials, the general result is still valid. To analyse the influence of these parameters, we compiled further models beside the two finite element simulations and the simple analytical approximation, and we varied the parameters which could have an influence. However, within the respective models, we found in the worst cases deviations of a factor of approximately three, but definitely not three orders of magnitude. That the epicuticle shows a higher Young's modulus than the underlying cuticle layers is in agreement with previous studies (Perez Goodwyn et al., 2006; Gorb, 2008).

The ultrastructural analyses of the arolium enables new insights into the fibrous organisation of the procuticle. The angled arrangement of the fibres in the proximal to distal orientation might lead to a higher tensile rigidity of the cuticle in this orientation than in the left to right orientation where the fibres are arranged perpendicularly to the epicuticle. This difference in tensile rigidity might explain why the cuticles showed almost no elongation in the longitudinal tensile test. In contrast to a previous study (Perez Goodwyn et al., 2006), fewer crosslinks between the fibres in the procuticle were found in our study. Extensive crosslinks between the fibres would increase the force needed to stretch the cuticle.

The method to determine the Young's modulus of biological superficial tissues by tensile testing is illustrated here for the first time, and refinements could enable us to achieve even more accurate values. For example, it would improve the accuracy of the method to know the exact thickness of the epicuticle and the other cuticle layers of the same stick insects as used in the tensile tests.

The determination of the Young's modulus of the epicuticle of *C. morosus* using tensile testing demonstrates the feasibility of analysing the mechanical properties of soft superficial biological tissues using this method followed by analytical evaluation and/or finite element simulation. The results support the assumption that smooth adhesive organs are covered with a very thin epicuticle of a high Young's modulus, which is supported by a fibrous procuticle, which equips the adhesive organs with the required softness needed for the conformation to a substrate. Biomimetic adhesive devices fabricated using this design principle would have the advantage of being less susceptible to contamination and abrasion.

## MATERIALS AND METHODS

### Study animals

Stick insects (*Carausius morosus*, Phasmatidae) were taken from the laboratory colony at the RWTH Aachen University, Institute for Biology II, Department Cellular Neurobionics.

### Light and scanning electron microscopy

Adult stick insects (565±95.1 mg, *N=*2) were stunned with CO_2_ and decapitated. The tarsi of all six legs were cut off and were fixed in 0.5% glutaraldehyde (Merck, Darmstadt, Germany) and 2% formaldehyde (Merck, Darmstadt, Germany) in PBS (AppliChem, Darmstadt, Germany) at room temperature for 24 h. The samples were post-fixed for 1 h in 1% osmium tetroxide (Sigma-Aldrich, St Louis, MO, USA) in PBS and thereafter washed in distilled water (three times for 15 min). The tarsi were dehydrated in an increasing ethanol series (30%, 50%, 70% each for 15 min) and subsequently stained for 1 h with 2% uranyl acetate solution (Merck, Darmstadt, Germany) in 70% ethanol (Carl Roth, Karlsruhe, Germany) in a dark environment. Subsequently, the samples were dehydrated in ethanol: 70% (three times), 80%, 90%, 96% and 100% (twice), each for 15 min, and washed twice for 30 min in propylene oxide (Serva, Heidelberg, Germany). They were then transferred to a mixture of propylene oxide and epoxy resin (Epon, Serva, Heidelberg, Germany) (1:1) from which the propylene oxide evaporated overnight. After washing in Epon (twice for 2 h), the samples were embedded in Epon, and the Epon was polymerised for 48 h at 60°C.

To analyse the layered structure of the cuticle of the arolium from embedded tarsi semi-thin sections of 1 μm thickness were cut longitudinally along the symmetrical plane of the tarsus using a Reichert OmU3 ultramicrotome (Reichert GmbH, Wien, Austria). The sections were attached to coverslips, stained with Methylene Blue (Riedel-de Haën, Seelze, Germany) for 30 s and washed with distilled water. Finally, the sections were dried on a heating plate and mounted with Depex (Serva, Heidelberg, Germany) on glass slides. Images were taken with a Motic BA 400 microscope (Motic Deutschland GmbH, Wetzlar, Germany) using a Moticam 3 and the Motic Images Plus 2.0 software at magnifications of ×100, ×400 and ×1000 in a 2048×1536 pixel format.

To analyse the detailed ultrastructure of the procuticle and the epidermis, semi-thin sections (1 μm) with epoxy resin etched off were prepared. For this, an Epon-embedded tarsus was sectioned longitudinally at the symmetrical plane of the tarsus with a Reichert OmU3 ultramicrotome using glass knives. The sections were picked up on coverslips and kept on a heating plate at 60°C for 4 h. Subsequently, the sections were treated with sodium methanolate solution (315 ml sodium methoxide, Fluka, Buchs, Switzerland; 185 ml methanol, Carl Roth, Karlsruhe, Germany; 100 ml toluene, Fluka, Buchs, Switzerland) in the absence of air for 6 min, methanol-toluene solution (1:1) for 5 min, 2× acetone (AppliChem, Darmstadt, Germany) for 5 min and were then washed in distilled water for 5 min in order to remove the resin. The samples were dehydrated with ethanol: 30, 50, 70, 90, 100% (twice), each step was performed for 5 min, and the samples were gradually transferred into hexamethyldisilazane (HMDS): 30, 50, 70, and 100% (twice), each step for 30 min, and finally the HMDS was left to evaporate overnight. The samples were mounted on holders using conductive double-sided adhesive tape (Plano GmbH, Wetzlar, Germany) and were gold coated with a sputter coater (Hummer; Technics, Alexandria, VA, USA) at 1 kV and 5 mA for 6 min. Observations were made with a Cambridge Stereoscan 200 (Cambridge Instruments, Somerville, MA, USA) at 10-15 kV acceleration voltage.

### Focused ion beam

To investigate the detailed ultrastructure of the cuticle, focused ion beam (FIB) treatment was used. Adult stick insects were stunned with CO_2_ and decapitated. The tarsi of all six legs were cut off and dehydrated in an increasing ethanol series [40%, 50%, 60%, 70%, 80%, 90%, 95%, 100% (twice)], each for 15 min, and were finally dried with a critical point dryer (Balzers CPD 030; Balzers Union Aktiengesellschaft, Lichtenstein, Germany). The samples were mounted on holders using conductive double-sided adhesive tape (Plano GmbH, Wetzlar, Germany) and coated with a gold layer of approximately 100 nm thickness with a sputter coater (S150B; Edwards, Crawley, West Sussex, UK) at 1.5 kV and 10 mA for 6 min.

Into an arolium, a rectangular window of 25×25 μm was cut with an incident angle of 30 deg to the surface of the arolium in the distal to proximal direction using a FIB Strata 205 (FEI, Hillsboro, OR, USA) at 20,000 pA. By this procedure, the cut went along the inclination angle of the fibres in the arolium in the distal to proximal direction. The cutting edges were polished at power of 1000 pA and coated with an approximately 50 nm thick tungsten layer using an *in situ* gas injection system to prevent electrostatic charge and damages resulting from Ga^+^ ions. Images were taken by a secondary electron detector at 50 pA in a 1024×954 pixel format.

### Tensile testing

**Fig. 7.**
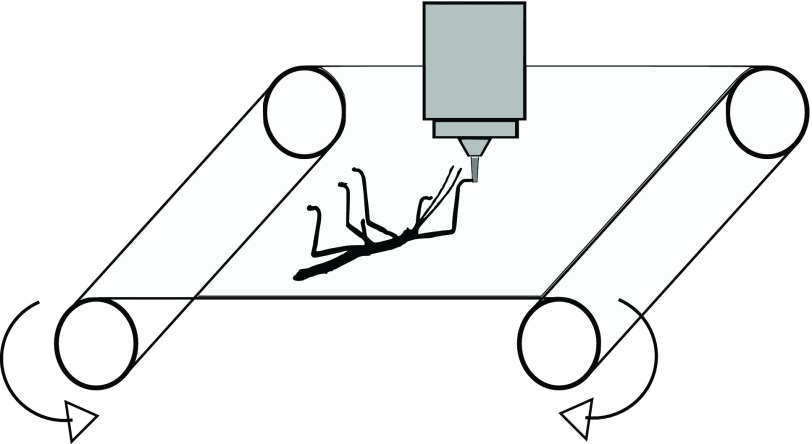
**Experimental set-up of the tensile tests.** A stick insect (black) adheres upside down on the underside of the latex membrane (light grey) and the objective of an epi-microscope (dark grey) is focused on one contact area between an arolium and the latex membrane. During the tensile tests, the latex membrane is stretched through rotation of the two bars and the elongations of the latex membrane and the arolium contact area are recorded.

Tensile testing of the arolia was performed using a measurement device built in-house (Fig. 7). Arolia of stick insects adhering to a latex membrane upside down were stretched through stretching of the latex membrane. Meanwhile, the elongation of the contact area between the arolium and latex membrane was recorded using an epi-illumination microscope (Axiophot 2, Carl Zeiss AG, Oberkochen, Germany) equipped with a ×10 LD Epiplan objective. The latex membrane was clamped onto two bars arranged parallel with a distance of 5 cm, which were connected through gears so that they rotated in opposite directions. The bars were rotated using a gear motor with a gear ratio of 3000:1 (Conrad Electronic, Hirschau, Germany) powered with 1.5 V, which was connected with one gear between the bars. The area of the membrane between the bars had a size of 39×50 mm and was stretched from the shorter edges of the latex membrane. The tensile test

device was mounted on an acrylic glass baseplate to enable transmitted light microscopy.

As a membrane, a latex condom with a thickness of 0.06 mm was used (Vitalis super thin, R&S consumer goods GmbH, Munich, Germany). The condom was washed with soap to remove the coating, both ends were cut off and it was cut open in length. Afterwards, it was spread, air dried and cut into 130×39 mm large pieces using a lasercutter (Epilog Zing 6030, power: 2%, speed 25%, Frequency 30 Hz).

The Young's modulus of the latex membrane used in the tensile tests was determined before the tensile tests. For this, a 56×39 mm large sample of a latex membrane was clamped on the lower side at a preload placed on a micro scales (JB1603-C/ FACT, Mettler-Toledo, Greifensee, Switzerland) and on the upper side at a pulling device in a way that only 40×39 mm in the middle of the membrane could be stretched. The pulling device was set to a condition in which the membrane was nearly fully extended but not stretched. In this condition, the micro scale was set to zero. From this condition the membrane was stretched using the pulling device and at intervals of 1 mm the stretching values on the micro scales were recorded during continuous stretching. The total elongation was 20 mm. The Young's modulus of the latex membrane was calculated using Eqn 1:
(1)
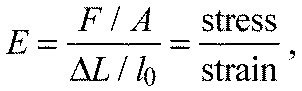

with *E* (MPa) being the Young's modulus of the latex membrane, *F* (N) being the force needed to extend the latex membrane, *A* (mm^2^) being the cross-sectional area of the latex membrane, Δ*L* (mm) being the elongation of the membrane and *l*_0_ (mm) being the length of the membrane at the beginning of the tensile test (40 mm). Plotting *F*/*A* against Δ*L*/*l*_0_ resulted in a force–strain curve of the latex membrane. Only the values providing a linear trend were included in a second diagram and a regression line was inserted. The functional equation of the regression line was calculated using Excel 2010, and the value of the slope was defined as the Young's modulus of the latex membrane.

Stick insects with a weight of approximately 14.8 mg (±5.2 mg, *N*=10) and a length of approximately 25 mm (±3 mm, *N*=10) were located in the tensile test device upside down on the latex membrane. The stick insects were aligned in a way that one arolium was orientated parallel or perpendicular to the pulling direction, preferably in the centre of the membrane. Depending on their alignment, the arolia were stretched in longitudinal or transversal directions. The stick insects were not fixed in their position, but were free to move on the latex membrane.

The latex membrane was stretched with a velocity of approximately 286 μm per second while a video of the elongation of the latex membrane and the contact area of an arolium was recorded with a digital camera (Moticam Pro 2850, Motic Deutschland GmbH, Wetzlar, Germany) in a 1360×1024 pixel format at 4 frames per second using the software Motic Images Plus 2.0 (Motic Deutschland GmbH).

During the tensile tests, the contact areas were kept in the view of the epi-microscope using the *xy*-stage, and the focus was continuously adjusted. The latex membrane was stretched to approximately 21.8% (±3.3%). This elongation is in the range in which the latex membrane stretches linear with the pulling force, according to the determination of the Young's modulus of the latex membrane. Single frames at the beginning and end of the tensile tests and at two time points in between, where the contact area of the arolium to the latex membrane was clearly visible, were chosen from the video recordings. Those frames were used to determine the elongation of the cuticle and the latex membrane. In these frames, the contact areas were measured using ImageJ (National Institutes of Health, Bethesda, MD, USA). Additionally, in the same frames, the distances between two prominent surface irregularities on the latex membrane, used as reference points, located in line with the direction of the stretching, were measured to analyse the elongation of the latex membrane.

### Evaluation of the tensile tests

Only experiments in which the contact area of the arolium was arranged in line or perpendicular to the stretching direction and in which the arolium did not slide on the latex membrane during the measurements were evaluated. The latter was fulfilled when the arolium contact area did not change its position towards reference points on the latex membrane. A further exclusion criterion was a large change in the contour of the arolium contact area during stretching.

To compute the Young's modulus of the epicuticle, a simple analytical approach, as well as two more sophisticated finite element models, were used.

For the analytical model, linear elastic behaviour of the materials under investigation was assumed, i.e. a linear relation of stress and strain. Furthermore the influence of the layers subjacent to the epicuticle and imperfections of the strain transmission due to geometric restrictions were neglected. When a force is applied onto a membrane, one has to distinguish the free latex membrane and the area where the latex membrane is coupled to the epicuticle. In the area of the free membrane one can calculate:
(2)
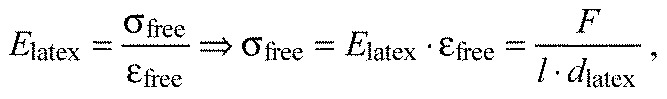

with *E*_latex_ being the Young's modulus of the latex membrane, σ_free_ being the stress tension (force per area) in the free latex membrane, ε_free_ being the strain (relative elongation) of the free latex membrane, *F* being the force acting on the free latex membrane, *l* being the width of the area under investigation and *d*_latex_ the thickness of the latex membrane. In the area of contact, the identical force *F* is transduced on a length, *l*. However, this force is due to the elastic deformation of the latex membrane and the epicuticle, which are in parallel. Thus, one can calculate the force in this contact area as:
(3)
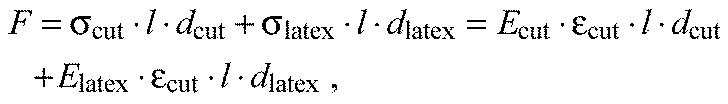

with σ_cut_ being the stress tension in the epicuticle, *d*_cut_ the thickness of the epicuticle, σ_latex_ the stress tension in the latex membrane, which stays in contact with the epicuticle and ε_cut_ being the strain in the contact zone of the latex membrane and arolium. The Young's modulus of the epicuticle is *E*_cut_. Inserting the expression for the force into Eqn 2, we obtain an expression for *E*_cut_, the Young's modulus of the epicuticle, to be
(4)
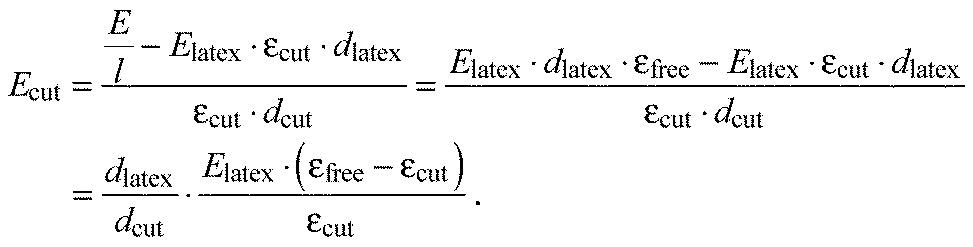



This is a rather vivid result as it can be seen that the ratio of the Young's modulus of the epicuticle and the supporting latex membrane is dependent on the thickness ratio and the relative reduction of the strain.

For a more detailed representation, we transferred the geometry of the tensile tests into finite element models. In the simulations, the latex membrane was stretched to the same extent as in the tensile tests. Now, the

**Fig. 8.**
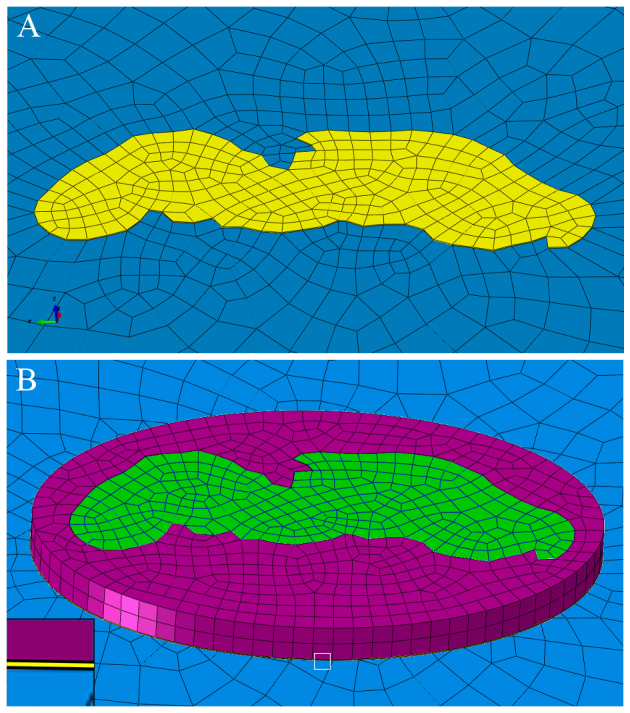
**CGI and CGII using the examples of the finite element models prepared to analyse the tensile test ‘transversal 3’.** (A) CGI consisting of a 400 nm thick cuticle (yellow) contacting the latex membrane (blue). The mesh of the cuticle is connected to that of the latex membrane. (B) CGII consisting of four parts: a 400 nm thick cuticle of the same geometry as the cuticle in CGI, which stays in contact with the latex membrane and on top of this a second 14.8 μm thick layer (green), which represents the layers of the contact zone in the arolium from the procuticle to the epidermis. These two cuticle layers are encircled by two surroundings (yellow and violet) showing the same material properties as the cuticle layers of the same thickness. The mesh of the surrounding of the thin cuticle layer is not connected to that of the latex membrane. The inset in image B shows the surrounding of the 400 nm thin cuticle layer in detail. The magnified area is highlighted by a white square.

Young's modulus inputted for the cuticle was adjusted until the elongation of the cuticle in the simulations coincided with the elongation of the cuticle during the tensile tests. The latex membrane in the models had the same geometry as in the measurements of 50×39×0.06 mm. The geometry of the cuticle was abstracted in two different ways.

- In CGI, the cuticle was assumed to be a layer of 400 nm thickness (Fig. 8), which corresponds to the thickness of the epicuticle (225 nm) plus an additional layer in which the thinnest fibres in the procuticle seem to be connected to each other and the epicuticle (Fig. 2). In this design of the cuticle, it was assumed that the fibres in the procuticle have no influence in the force needed to stretch the arolium.
- In CGII, the cuticle had a total thickness of 8.37 to 15.2 μm, which corresponds to the thickness of all layers in the arolium from the epicuticle to the epidermis (Fig. 8). The total thickness was varied because of the differences in size of the arolia of the different stick insects. The contact zone is composed of two layers with different Young's moduli: a 400 nm thin layer, connected to the latex membrane and a thicker cuticle on top, i.e. the rest of the total thickness, which represented the cuticle layers in the arolium from the procuticle to the epidermis. Additionally, the thin and the thick cuticle layers were surrounded by layers of the same thickness. The surrounding layers were 10% larger than the arolium contact area (left to right), and the length of the surrounding layers (proximal to distal) amounted to two-thirds of their width. With this additional material, it should be considered that the cuticle around the arolium contact area also got squeezed or stretched in the tensile tests. The layer surrounding the thin cuticle layer had no connection to the latex membrane. In the simulations, all cuticles were placed on the latex membrane at the same geometry as that in the tensile tests, as determined by microscopy.

For the nodes at the sides of the latex membrane, from which the latex membrane was stretched, the degrees of freedom were reduced so that these nodes could only move in the direction of the stretching. For all nodes in the models, the displacements perpendicular to the plane of the latex membrane were set to zero, because otherwise the large deformations of the latex membrane could not be calculated. On all nodes at both sides of the latex membrane, where the stretching occurred, a displacement equal to the percentage elongation of the latex membrane calculated for the individual tensile tests was applied.

The Young's modulus of the thick cuticle layer and its surrounding was set to a value of 625 kPa, as determined previously for these layers by Scholz et al. (Scholz et al., 2008). The Young's modulus of the 400 nm thin cuticle in both models and in CGII also the surroundings of the 400 nm thin cuticle layers was initially set to an assumed value. At the beginning and end of the stretching, the distance between two nodes at the outer corners of the cuticle, which lay in line with the direction of the stretching and were in contact with the latex membrane, were measured.

To determine the correct Young's modulus of the epicuticle, the Young's modulus inputted for this layer was adjusted until the distance between the nodes at the outer corners of the cuticle showed the same elongation in the finite element models as in the tensile tests.

The Young's modulus of the latex membrane was set to 1.2 MPa, as determined previously. The Poisson's ratio of the latex membrane and the cuticles was set to 0.3. The Poisson's ratio of the cuticles was not set to a higher value, because the Poisson's ratio of the arolium in proximal to distal direction might be negative (Dirks et al., 2012).

Four tensile tests in the transversal direction were analysed by finite element simulation as described above using the software Abaqus 6.11 (Dassault Systemes, Vélizy-Villacoublay Cedex, France). All elements in the finite element models had the same element type of C3D8R, which is an 8-node brick with reduced integration and hourglass control.

For meshing, first the cuticles were meshed in a way that their contours were subdivided into 75 elements. Inside the contours, the cuticles were filled out with elements of the same size. Occasionally, minor changes to these subdivisions into elements had to be used, because the automatic meshing failed with the subdivisions mentioned above. The meshes of the cuticles were transferred to the latex membrane, in those areas where the cuticles contacted the latex membrane. The latex membrane, which had no contact with the cuticle, was meshed in such a way that the elements got bigger from the area where it contacted the cuticle to the edges of the latex membrane, up to an edge length of 2 mm. In CGI, the nodes of the thin cuticle layer and the latex membrane contacting each other were merged.

The outer contours of the surroundings in CGII were subdivided into 75 elements. The subdivision into elements of the inner contours of the surroundings was adopted from the contours of the cuticle layers. In CGII, the nodes of the latex membrane and the nodes of the thin and the thick cuticles contacting each other were merged. The nodes of the surroundings of CGII were not merged with the other geometries, but the perpendicular surfaces at the inner contours of the surroundings and the perpendicular surfaces at the contours of the cuticles were connected through the tie constraint. Besides, the upper surfaces of the thin surroundings and the lower surfaces of the thick surroundings were connected by this constraint. Using this procedure, the nodes on the lower surface of the thin cuticle surrounding and the adjacent nodes on the top surface of the latex membrane were not connected to each other.

In all models, the latex membrane had a thickness of one element, as well as the cuticles with a thickness of 400 nm and in CGII the surroundings of the 400 nm thick cuticles also had a thickness of one element. The thick cuticle in CGII and its surrounding had a thickness of one or two elements, depending on their thickness in reference to the edge length of their elements in the plane of the latex membrane.

Additionally, the Young's modulus of the epicuticle was estimated using seven further tensile tests. For these tensile tests, no finite element models were prepared, but the tensile tests were analysed using the results of the finite element simulations of the first four tensile tests. For this, the Young's moduli determined in the finite element simulations were plotted individually for each cuticle geometry against the percentage elongations of the cuticles in reference to the percentage elongations of the latex membrane. The resulting data points were connected with linear regression lines, and the functional equations of the regression lines were calculated with Excel 2010. These functional equations were used to calculate the Young's moduli of the epicuticles of the further seven tensile tests using their percentage elongation of the cuticle in reference to the percentage elongation of the latex membrane.

## Supplementary Material

Supplementary Material
