# Supplementary material for: Determination of the Young's modulus of the epicuticle of the smooth adhesive organs of Carausius morosus using tensile testing
Source: J Exp Biol. 2014 Oct 15;217(20):3677–87. doi: 10.1242/jeb.105114 (PMC4198382; doi:10.1242/jeb.105114)
Supplement: Supplementary Material [file supp_217_20_3677__index.html]

Determination of the Young's modulus of the epicuticle of the smooth adhesive organs of Carausius morosus using tensile testing — Supplementary Material 

# Determination of the Young's modulus of the epicuticle of the smooth adhesive organs of *Carausius morosus* using tensile testing

## JEB105114 Supplementary Material

**Files in this Data Supplement:**

- **Supplementary Material**
