# Supplementary material for: Determination of the Young's modulus of the epicuticle of the smooth adhesive organs of Carausius morosus using tensile testing
Source: J Exp Biol. 2014 Oct 15;217(20):3677–87. doi: 10.1242/jeb.105114 (PMC4198382; doi:10.1242/jeb.105114)
Supplement: Supplementary Material [file supp_217.20.3677_JEB105114.pdf]

Supplementary material

Table S1: Determination of the Young´s modulus of the latex membrane used in the tensile tests. N=newton, t_0=time of the beginning of the stretching of the membrane. Only the values providing a linear trend are shown.

**measurement**	**force needed to extend the membrane (F) [N]**	**cross sectional area (A) [mm²]**	**elongation of the membrane (ΔL) [mm]**	**length of the membrane at t_0 (l_0) [mm]**	**F/A**	**ΔL/ l_0**	**determined Young´s modulus [MPa]**
**1**	0.000	2.34	0	40	0	0	
	0.053	2.34	1	40	0.022	0.025	
	0.133	2.34	2	40	0.057	0.05	
	0.216	2.34	3	40	0.092	0.075	
	0.281	2.34	4	40	0.120	0.1	
	0.358	2.34	5	40	0.153	0.125	
	0.420	2.34	6	40	0.180	0.15	
	0.477	2.34	7	40	0.204	0.175	
	0.547	2.34	8	40	0.234	0.2	**1.19**
**2**	0.000	2.34	0	40	0	0	
	0.049	2.34	1	40	0.021	0.025	
	0.124	2.34	2	40	0.053	0.05	
	0.200	2.34	3	40	0.085	0.075	
	0.280	2.34	4	40	0.120	0.1	
	0.348	2.34	5	40	0.149	0.125	
	0.412	2.34	6	40	0.176	0.15	
	0.474	2.34	7	40	0.203	0.175	
	0.544	2.34	8	40	0.233	0.2	**1.189**
**3**	0.000	2.34	0	40	0	0	
	0.055	2.34	1	40	0.024	0.025	
	0.141	2.34	2	40	0.060	0.05	
	0.208	2.34	3	40	0.089	0.075	
	0.282	2.34	4	40	0.120	0.1	
	0.358	2.34	5	40	0.153	0.125	
	0.422	2.34	6	40	0.180	0.15	
	0.488	2.34	7	40	0.208	0.175	
	0.556	2.34	8	40	0.238	0.2	**1.2062**
**average**							**1.1953**
**standard deviation**							**0.0076**

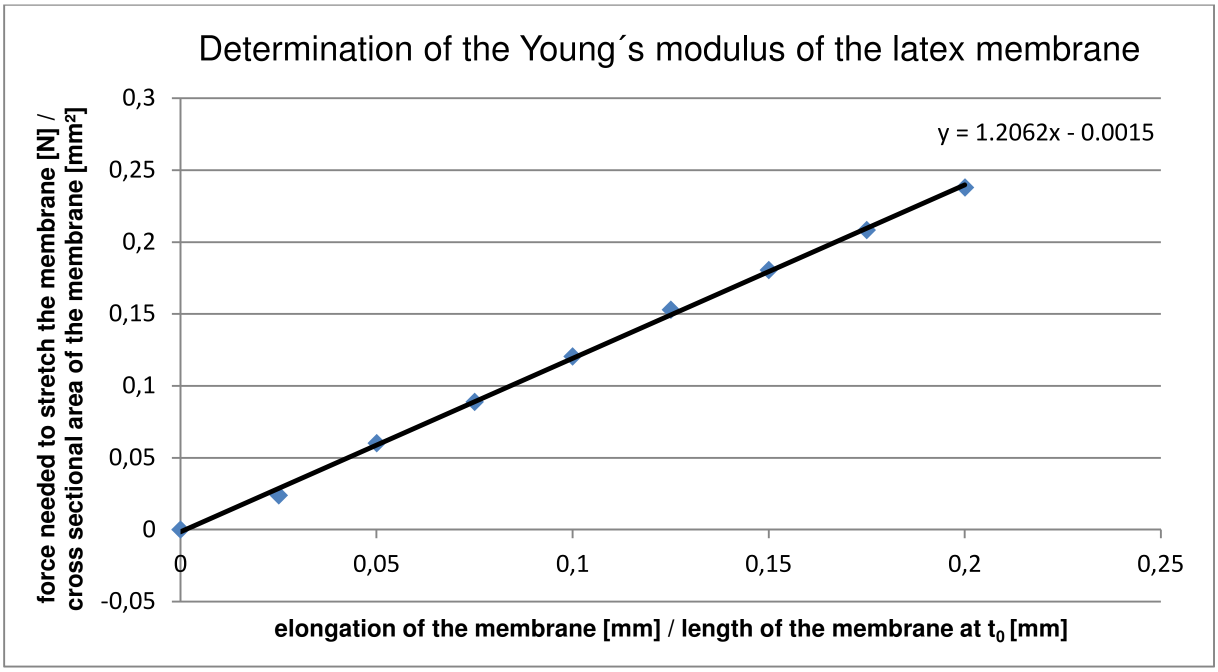

Fig. S1: Exemplary tensile test performed to determine the Young´s modulus of the latex membrane. Only the data points providing a linear trend are shown. The data points were connected with a linear regression line. In the top right corner the functional equation of the regression line is given. The slope of the line denotes the Young´s modulus determined for the latex membrane in this tensile test in megapascal.

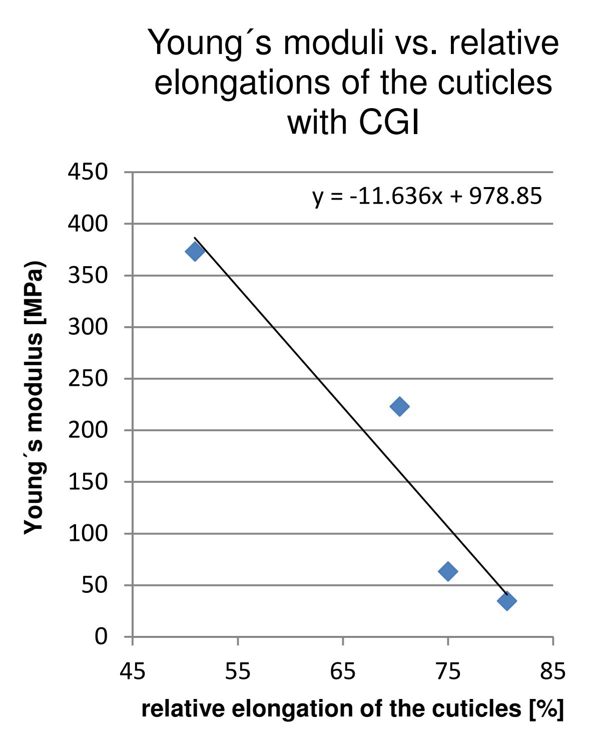

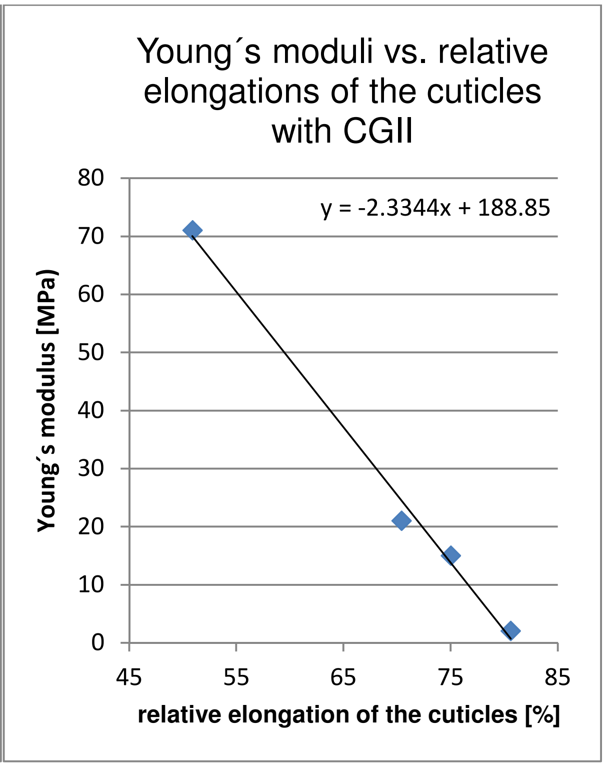

Fig. S2: In the finite element simulations determined Young´s moduli plotted against the percentage elongation of the cuticles in reference to the percentage elongation of the latex membrane. In both diagrams linear trend lines were inserted and their functional equations are shown in the top right corners of the diagrams. The functional equations were used to calculate the Young´s moduli of the cuticles of the stick insects used in the further seven tensile tests.
